# Work Characteristics of Acute Care Surgeons at a Swiss Tertiary Care Hospital: A Prospective One-Month Snapshot Study

**DOI:** 10.1007/s00268-021-06350-7

**Published:** 2021-10-22

**Authors:** Claudine Di Pietro Martinelli, Tobias Haltmeier, Joël L. Lavanchy, Stéphanie F. Perrodin, Daniel Candinas, Beat Schnüriger

**Affiliations:** Department of Visceral Surgery und Medicine, lnselspital, Bern University Hospital, University of Bern, Bern, Switzerland

## Abstract

**Background:**

Multiple acute care surgery (ACS) working models have been implemented. To optimize resources and on-call rosters, knowledge about work characteristics is required. Therefore, this study aimed to investigate the daily work characteristics of ACS surgeons at a Swiss tertiary care hospital.

**Methods:**

Single-center prospective snapshot study. In February 2020, ACS fellows prospectively recorded their work characteristics, case volume and surgical case mix for 20 day shifts and 16 night shifts. Work characteristics were categorized in 11 different activities and documented in intervals of 30 min. Descriptive statistics were applied.

**Results:**

A total of 432.5 working hours (h) were documented and characterized. The three main activities ‘surgery,’ ‘patient consultations’ and ‘administrative work’ ranged from 30.8 to 35.9% of the documented working time. A total of 46 surgical interventions were performed. In total, during day shifts, there were 16 elective and 15 emergency interventions, during night shifts 15 emergency interventions. For surgery, two peaks between 10:00 a.m.–02:00 p.m. and 08:00 p.m.–11:00 p.m. were observed. A total of 225 patient were consulted, with a first peak between 08:00 a.m. and 11:00 a.m. and a second, wider peak between 02:00 p.m. and 02:00 a.m.

**Conclusion:**

The three main activities ‘surgery,’ ‘patient consultations’ and ‘administrative work’ were comparable with approximately one third of the working time each. There was a bimodal temporal distribution for both surgery and patient consultations. These results may help to improve hospital resources and on-call rosters of ACS services.

## Introduction

Conceived in 2003 in Northern America, the concept of acute care surgery (ACS) is globally evolving. Encompassing emergency general surgery (EGS), trauma surgery and surgical critical care, the implementation of an ACS service has shown to improve efficacy in the management of various surgical emergencies [1–4].

Recognizing the specific needs, time sensitiveness of treatment, and complexity of patients with abdominal surgical emergencies, an ACS service has been established at Bern University Hospital in 2016. Bern University Hospital is a tertiary care facility with a catchment area of approximately one million people. Annually, around 750 EGS procedures are carried out at Bern University Hospital. Within the Department of Visceral Surgery and Medicine, the ACS team takes responsibility for 24 h EGS interventions and consultations Monday through Friday. On weekends, additional staff from other surgical subspecialties (i.e., colorectal, hepatobiliary) from the Department of Visceral Surgery and Medicine are taking calls.

In addition to EGS procedures, the ACS team does consultations for patients with acute traumatic or non-traumatic abdominal disease in the emergency department (ED), intensive care unit (ICU), on regular wards and outpatient clinics of Bern University Hospital. Moreover, the ACS team closely follows critically ill patients with abdominal diseases on the ICU daily, in collaboration with intensive care specialists. Elective procedures, such as cholecystectomies, hernia repairs, colorectal procedures for benign diseases (i.e. subsequent colostomy takedown) and planned re-laparotomies, are also part of the daily routine of the ACS team.

Whereas an increasing number of ACS working models have been implemented and further developed worldwide [5–9], no data are available about the daily practice of ACS surgeons in Switzerland. To optimize hospital resources and on-call rosters, knowledge about work characteristics and distribution is required. Therefore, this study aimed to investigate for the first time the daily work characteristics of ACS surgeons at a Swiss tertiary care hospital, taking into account both the characteristics and timely distribution of the work activity.

## Material and methods

At the Department of Visceral Surgery and Medicine at Bern University, there is a designated Acute Care Surgery Team in place, including three ACS fellows and four surgical residents that work under the supervision of an attending surgeon specialized in the field of ACS. These fellows are board-certified general surgeons and are in their further training to become specialists in visceral surgery. The fellows from the entire department rotate into the ACS Team for at least 6 months to further train and improve their skills and knowledge on the broad care of acute surgical patients. A senior attending ACS surgeon very closely supervises and teaches them. In addition, the organ specific specialists (upper gastrointestinal surgery, hepatobiliary surgery, etc.) are consulted as needed.

ACS fellows work in a two-shift system. A day shift lasts from 07:30 a.m. to 05:00 p.m. (9.5 h) and the night shift from 05.00 p.m. until 07.30 a.m. (14.5 h), respectively. Attendance time is mandatory during the day shift, whereas ACS fellows are on-call at night.

From February 1 to February 29, 2020, three ACS fellows prospectively documented detailed data regarding the characteristics of their work, case volume and surgical case mix in intervals of 30 min for a total of 20 day shifts and 16 night shifts.

Collected data were grouped into three main categories and 11 subcategories: Surgery (elective and emergency surgery), Patient consultations (on regular wards, ICU, ED or outpatient clinic) and administrative work (morning reports, board participation, research, teaching and office work). Specifically during the night shift, the time being solely on-call without presence in the operating room (OR) or patient consultations or administrative work was documented as such.

Descriptive statistics were applied. Categorical variables are documented as numbers and percentages, and continuous variables including times are given as median and ranges.

## Results

In February 2020, during 20 day and 16 night shifts, a total of 432.5 working hours (*h*), (198.5 h during day shifts, 234.0 h during night shifts) were prospectively documented and characterized by a total of three ACS fellows. The activity profiles, stratified according to day or night shifts, are outlined in Table 1. The three main activities ‘surgery,’ ‘patient consultations’ and ‘administrative work’ were comparable with 30.8–35.9% of the documented working time. Additionally, at night shifts, the fellows were in-house for a median of 8.3 h (range 5.0–16.5 h) and on-call (without specific activity) for a median of 6.5 h (range 1.5–9.5 h).

**Table 1:** Activity profile of ACS fellows

	Total			Day shift			Night shift		
	Median*	Range	(%)	Median*	Range	(%)	Median	Range *	(%)
Surgery	3.3	0.5–11.0	(33.3)	2.5	0.5–11.0	(27.8)	3.5	0.5–6.0	(35.0)
Elective surgery	3.5	0.5–4.5		3.5	0.5–4.5		–	–	
Emergency surgery	2.5	0.5–7.0		1.5	0.5–7.0		3.5	0.5–6.0	
Patient consultations	3.5	0.5–6.5	(35.9)	3.3	0.5–6.5	(36.1)	3.5	0.5–6.5	(35.0)
Regular ward	2.0	0.5–3.0		2.0	0.5–3.0		2.3	0.5–2.5	
ED and shockroom	1.0	0.5–3.5		1.0	0.5–3.5		1.0	1.0–3.5	
Outpatient clinic	1.0	0.5–4.0		1.0	0.5–4.0		–	–	
ICU	0.5	0.5–3.5		0.5	0.5–3.5		0.8	0.5–3.5	
Administrative work	3.0	0.5–7.5	(30.8)	3.3	0.5–7.5	(36.1)	3.0	1.0–7.5	(30.0)
Office work	2.5	0.0–5.5		2.5	0.0–5.5		2.5	0.5–5.5	
Research	1.8	1.0–3.5		1.3	1.0–3.5		2.0	1.0–3.5	
Teaching	1.0	1.0–1.0		1.0	1.0–1.0		1.0	1.0–1.0	
Board	1.0	0.5–1.5		1.0	0.5–1.5		0.5	0.5–0.5	
On-call time	–	–	(–)	–	–	(–)	6.5	1.5–9.5	(–)

*Medians are reported as hours per day (24 h)

*ACS*: Acute Care Surgery; *ED*: Emergency Department; *ICU*: Intensive Care Unit

**Fig. 1:**
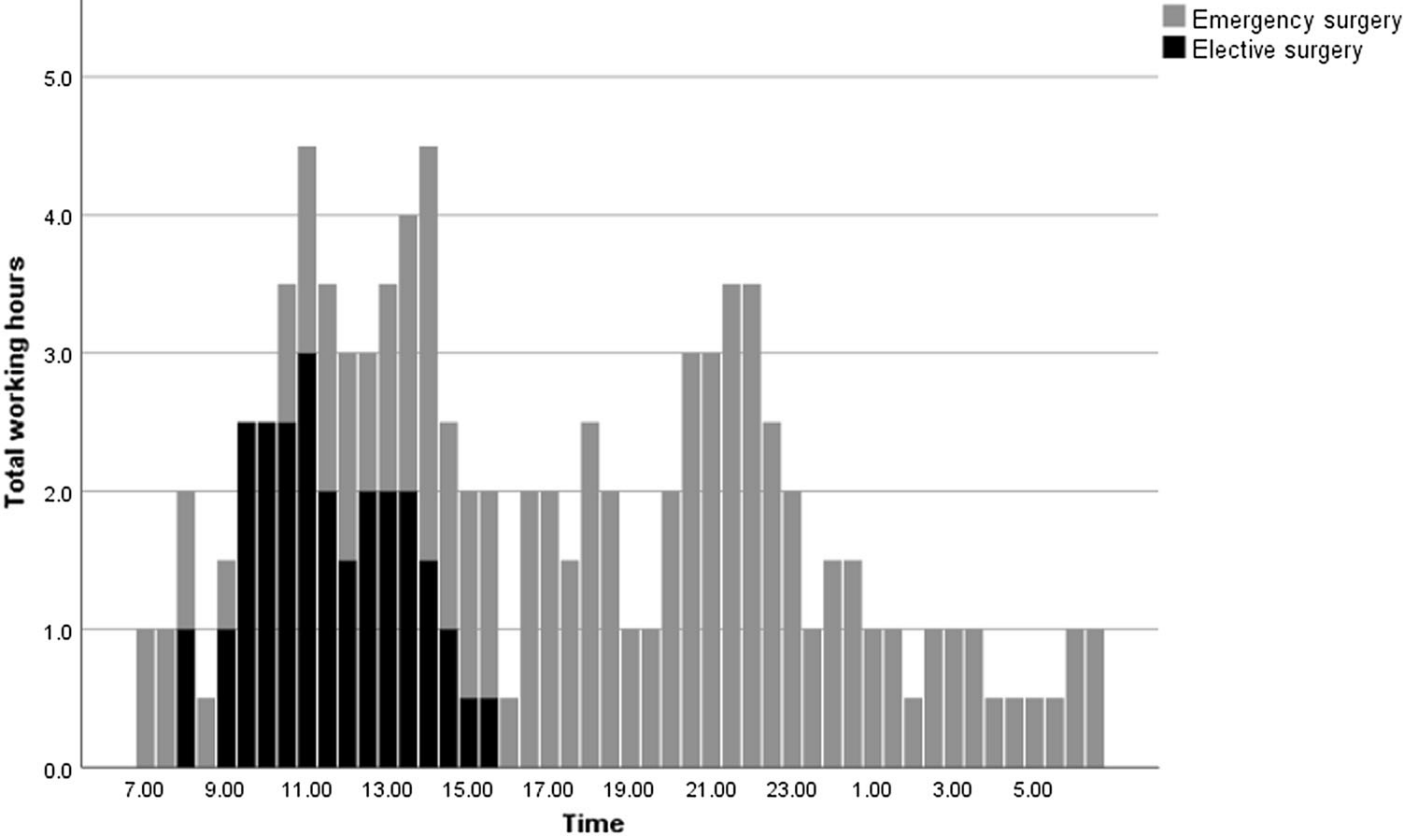
Temporal distribution of surgery

A total of 46 surgical interventions were performed by the three ACS fellows during the documented study period of 20 days and 16 nights. During the day shifts, there were 16 elective and 15 emergency interventions and 15 emergency interventions during night shifts. These surgical interventions are outlined in Table 2. Most frequent *emergency* surgical interventions were diagnostic laparoscopies and laparotomies for intestinal perforation (*n* = 7), laparoscopic appendectomies (*n* = 6) and laparoscopic cholecystectomies (*n* = 5). Most frequent *elective* surgical interventions included hernia surgery (*n* = 6) and laparoscopic cholecystectomies (*n* = 3). Regarding temporal distribution of surgery, there was a first peak between 10:00 a.m. and 02:00 p.m. and a second peak between 08:00 p.m. and 11:00 p.m. (Fig. 1).

**Table 2:** Surgical case mix

	Total		Day shift				Night shift	
			Elective		Emergency		Emergency	
	*n*	(%)	*n*	(%)	*n*	(%)	*n*	(%)
Cholecystectomy	8	(17.4)	3	(6.5)	2	(4.4)	3	(6.5)
Intestinal perforation	7	(15.3)	–	(–)	2	(4.4)	5	(10.8)
Appendectomy	6	(13.0)	–	(–)	4	(8.6)	2	(4.4)
Hernia surgery	6	(13.0)	6	(13.0)	–	(–)	–	(–)
i.v.-ports and catheters	5	(10.9)	2	(4.4)	3	(6.4)	–	(–)
Intestinal obstruction	4	(8.7)	–	(–)	1	(2.2)	3	(6.5)
Proctology	3	(6.5)	1	(2.2)	2	(4.4)	–	(–)
Mesenteric ischemia	1	(2.2)	–	(–)	–	(–)	1	(2.2)
Other (including trauma)	6	(13.0)	4	(8.7)	1	(2.2)	1	(2.2)
Total	46	(100)	16	(34.8)	15	(32.6)	15	(32.6)

**Fig. 2:**
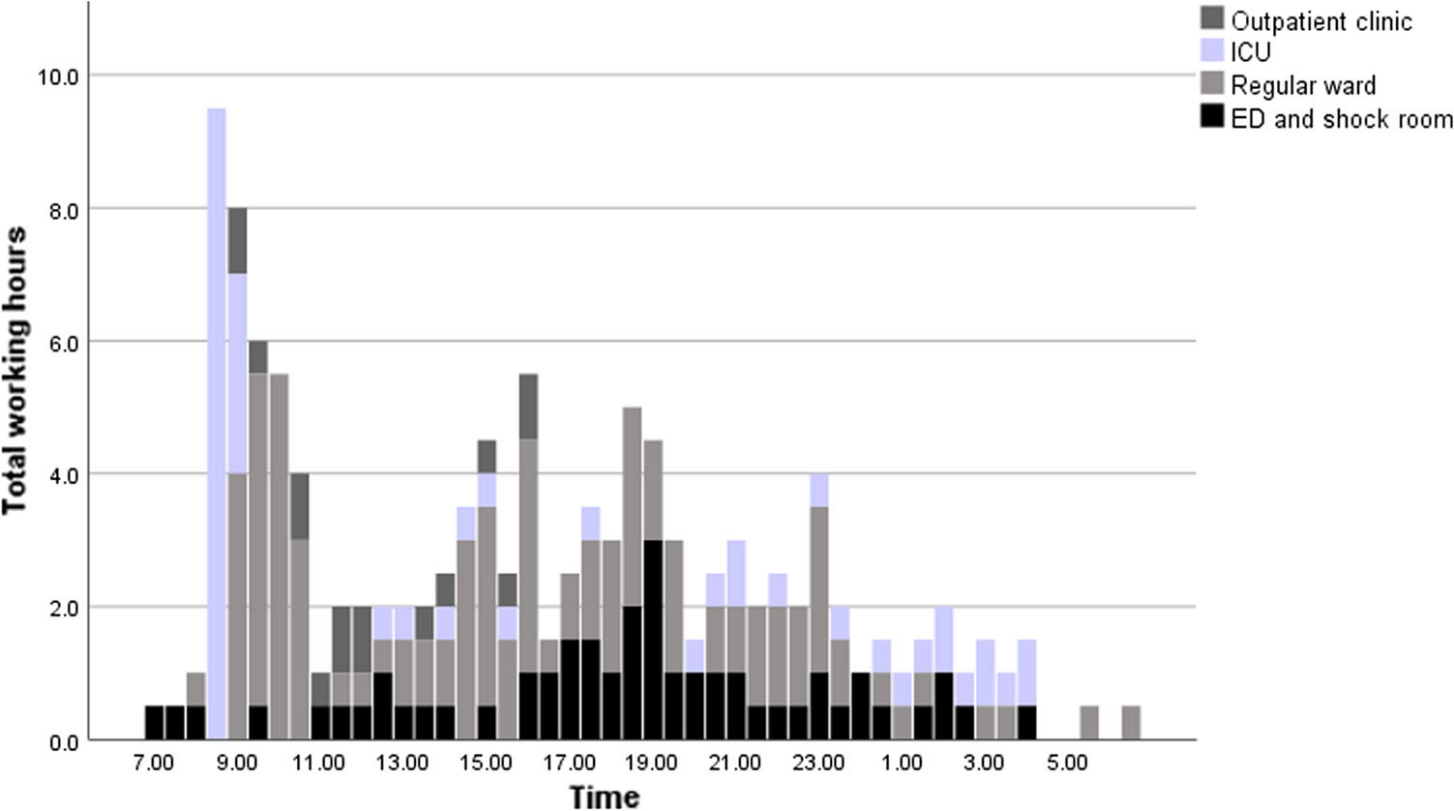
Temporal distribution of patient consultations

During the study period, a total of 225 patient consultations were performed. Thereof, 124 (55%) consultations were carried out during the day shifts and 101 (45%) consultations during the night shifts. Of the 225 patient consultations, 92 (41%) were performed on surgical, orthopedic and various nonsurgical wards, 68 (30%) in the ED and 65 (29%) in the outpatient clinic. Figure 2 demonstrates the temporal distribution of patient consultations. The daily scheduled ICU and ward rounds resulted in a first sharp peak of consultations between 08:00 a.m. and 11:00 a.m. Moreover, there was a second wider peak between 02:00 p.m. and 02:00 a.m. with predominantly consultations in the ED and regular wards. Between 04:00 a.m. and 08:00 a.m. a very low number of patient consultations were performed.

### Day shift (07:30 a.m. to 05:00 p.m.)

During the day shift of a total of 9.5 h, surgery accounted for a median of 2.5 h (range 0.5–11.0 h). These included 3.5 h (range 0.5–4.5 h) elective and 1.5 h (range 0.5–7.0 h) EGS procedures. Patient consultations accounted for a median of 3.3 h (range 0.5–6.5 h). These comprised of consultations on the wards [2.0 h (range 0.5–3.0 h)], ED and shock room [1.0 h (range 0.5–3.5 h)], outpatient clinic [1.0 h (range 0.5–4.0 h)] and ICU [0.5 h (range 0.5–3.5 h)]. Administrative work accounted for a median of 3.3 h (range 0.5–7.5 h).

These included office work [2.5 h (range 0.0–5.5 h)], research [1.3 h (range 1.0–3.5 h)], morning reports and tumor or trauma board participations [1.0 h (range 0.5–1.5 h)] and teaching [1.0 h (range 1.0–1.0 h)].

### Night shift (05:00 p.m. to 07:30 a.m.)

During the night shift of a total of 14.5 h, the ACS fellows were working in-house for a median of 8.3 h (range 5.0–16.5 h) and on-call without specific activity for a median of 6.5 h (range 1.5–9.5 h). Of working time at night, emergency surgery accounted for a median of 3.5 h (range 0.5–6.0 h). No elective interventions were performed at night. Patient consultations accounted for a median of 3.5 h (range 0.56.5 h). These included consultations on the wards [2.3 h (range 0.5–2.5 h)], ICU [0.8 h (range 0.5–3.5 h)] and ED and shock room [1.0 h (range 1.0–3.5 h)]. No ‘elective’ consultations in the outpatient clinic were performed during the night shift. Administrative work accounted for a median of 3.0 h (range 1.0–7.5 h). These included morning reports and tumor or trauma board participations [0.5 h (range 0.5–0.5 h)], research [2.0 h (range 1.0–3.5 h)], teaching [1.0 h (range 1.0–1.0 h)] and office work [2.5 h (range 0.5–5.5 h)].

## Discussion

This study investigated the daily work characteristics of ACS surgeons at a Swiss tertiary care hospital, taking into account both the characteristics and temporal distribution of the activities. Currently, no similar investigations are available. The three main activities ‘surgery,’ ‘patient consultations’ and ‘administrative work’ were comparable taking up approximately one third of the documented working time each. There was a bimodal temporal distribution for both surgery and patient consultations. Between 04:00 a.m. and 08:00 a.m. workload settled due to reduced patient consultations and surgical interventions.

In 2006, ACS has been implemented in the USA with great success regarding clinical outcomes, optimization of acute patient’s care, research knowledge and surgical satisfaction [10–14]. However, little data are available on the details of the daily work characteristics of ACS fellows [15, 16].

In February 2020, a snapshot of working practice before the COVID-19 pandemic could be achieved. Of note, the number of surgical interventions was somewhat below the calculated median, taking into account the overall 744 emergent abdominal surgical interventions that were performed in 2020 at Bern University Hospital. However, the number of EGS interventions was comparable to other centers with similar size and characteristics [17, 18].

In contrast to the number and characteristics of EGS interventions, little is known about the amount of patient consultations and required administrative work. In the current study, pre- and postoperative care, as well as the management of non-operatively treated patients, occupied a third of the overall working time. This considerable amount of non-operative working time reflects the ACS model, where the assessment of acute patients or close follow-up of conservatively treated patients plays a major role. Moreover, the presence of the ACS surgeon in the ICU is of great importance to provide optimal interdisciplinary treatment of critically ill surgical patients [19–21].

Administrative work is considerable and is related to various tasks such as billing and insurance-related paperwork, as well as for medico-legal requirements [22, 23]. Careful monitoring of the development of the time-consuming administrative component of our work is required to avoid further increase. The bimodal distribution of surgery is related to the elective caseload at day shifts, the operating room resources and prioritization of emergency interventions. At Bern University Hospital, there is a designated OR for patients with an expected serious adverse outcome if not operated on within six hours. The remaining patients undergo surgical intervention after or in between the elective surgical procedures. A considerable amount of emergent surgical interventions is performed between 08:00 p.m. and 11:00 p.m. This needs to be taken into account when planning and scheduling teams of ACS teams. Patients’ consultations first peaked in the morning due to scheduled daily rounds on ICU and wards. A second wider peak was found and is reflecting a more continuous demand for acute surgical assessment and care from 02:00 p.m. to 02:00 a.m. with predominantly consultations in the ED, shockroom and wards. This bimodal distribution underlines the importance of the presence of ACS fellows for the evaluation of patients throughout the entire institution at any time. The timely acute assessment and troubleshooting of patients are of paramount importance and has a direct impact on patients’ outcomes [10, 24–27].

In 2015, Pottenger et al. analyzed frequency counts and work relative value units generated for specific codes to characterize the average trauma and emergency surgeon's work experience over time in the USA.

These investigators found that acute care surgery consists of 40% surgical and 60% cognitive work [28]. Similar to these findings, the current prospective snapshot study showed that a considerable part of the daily routine of acute care surgeons consists of non-procedural work, whether this amount of non-operative skills is larger than in other surgical subspecialties is uncertain and warrants further investigations.

## Conclusion

At the investigated Swiss tertiary care center, the workload of ACS surgeons includes mainly EGS interventions, pre- and postoperative patient consultations, non-operative patient management and administrative work. Both broad general surgical and cognitive skills should be implemented into adapted training programs to meet the unique and challenging requirements of acute care surgery.
